# Clinically Relevant Anti-Inflammatory Agents for Chemoprevention of Colorectal Cancer: New Perspectives

**DOI:** 10.3390/ijms19082332

**Published:** 2018-08-08

**Authors:** Altaf Mohammed, Nagendra Sastry Yarla, Venkateshwar Madka, Chinthalapally V. Rao

**Affiliations:** 1 Current Address: Chemopreventive Agent Development Research Group, Division of Cancer Prevention, National Cancer Institute, Rockville, MD 20850, USA; altaf.mohammed@nih.gov; 2 Center for Chemoprevention and Drug Development, University of Oklahoma Health Sciences Center, Oklahoma City, OK 73104, USA; sastryyn@gmail.com (N.S.Y.); Venkateshwar-Madka@ouhsc.edu (V.M.)

**Keywords:** colon cancer, chemoprevention, anti-inflammatory agents, NSAIDs

## Abstract

Substantial efforts are underway for prevention of early stages or recurrence of colorectal cancers (CRC) or new polyp formation by chemoprevention strategies. Several epidemiological, clinical and preclinical studies to date have supported the chemopreventive potentials of several targeted drug classes including non-steroidal anti-inflammatory drugs (NSAIDs) (aspirin, naproxen, sulindac, celecoxib, and licofelone), statins and other natural agents—both individually, and in combinations. Most preclinical trials although were efficacious, only few agents entered clinical trials and have been proven to be potential chemopreventive agents for colon cancer. However, there are limitations for these agents that hinder their approval by the food and drug administration for chemoprevention use in high-risk individuals and in patients with early stages of CRC. In this review, we update the recent advancement in pre-clinical and clinical development of selected anti-inflammatory agents (aspirin, naproxen, sulindac, celecoxib, and licofelone) and their combinations for further development as novel colon cancer chemopreventive drugs. We provide further new perspectives from this old research, and insights into precision medicine strategies to overcome unwanted side-effects and overcoming strategies for colon cancer chemoprevention.

## 1. Introduction

Colorectal cancer (CRC), is the third most common cancer among men and women, and is a preventable cancer once identified at early stages through different chemoprevention strategies [1,2]. In 2018, over 140,000 new cases are expected and over 50,000 people die of CRC in US alone while globally over 2.2 million new cases and over 1.1 million deaths are expected by 2030 [1,3,4]. Approximately one in eighteen individuals will develop CRC over their lifetime and 40% will die within five years of diagnosis, mainly due to diagnosis at a late stage [5,6]. Most CRC cases are reported in the developed countries, like USA. Chronic inflammation is one of the risk factors for colorectal cancers [7]. Diet and life-style changes, along with genetic and hereditary risk factors, play an important role in relatively long period of time leading to progression of precancerous stages to CRC [8]. Obesity, alcohol, aging, hypertriglyceridemia and smoking are known risk factors. CRC, by disease itself is extensively studied preclinically and at clinical level [9,10]. Several previous studies suggested that NSAIDs can prevent risk factors-associated CRC [11,12].

Intestinal tumorigenesis is a complex process, in which many tumor suppressors and oncogenic mediators are in involved [13,14,15]. Adenomatous polyposis coli (APC) is mutated in more than 80% of patients with intestinal cancers including an autosomal dominant inherited condition familial adenomatous polyposis (FAP) [13,14,15].

CRC development, like many other cancers is a slow process that takes several years for initiation to precursor lesion formation to progression into adenoma, carcinoma, invasion and metastasis [16,17]. There will be ample opportunity to intervene at the precursor/adenoma stage thereby preventing the progression into invasive carcinoma by screening and chemopreventive interventions [18,19,20]. Due to existing technologies and significant advances in the early detection of CRC by colonoscopy or identifying high risk population at forefront is driving the science towards developing appropriate chemoprevention strategies [18,19].

Over past three decades, extensive efforts are diverted towards colon cancer chemoprevention especially at the preclinical level. Targeting tumor-associated inflammation is one of the strategies for colon cancer prevention [7]. Several non-steroidal anti-inflammatory drugs (NSAIDs) have been explored as chemopreventive agents for chemoprevention of colorectal cancers [12,21]. Majority of the agents belonging to natural, semi-synthetic or synthetic class are studied in in vitro and in vivo animal studies. Although several of these agents showed good to profound effects, only a handful of them entered clinical trials. The main reason for this is the high bar for toxicity in the cancer chemoprevention trails. Also, significant time and appropriate cohorts’ availability also delays chemoprevention trials. In view of the toxicity limitations, new strategies are being employed in in vivo studies to overcome potential side-effects while retaining the efficacy of the drugs. Use of low-dose combination of drugs that target complementary pathways has been in use for long time now. Other approaches such as alternate dosing, intermittent scheduling or short-term frequent dosing, alternative route of drug administration etc. are being explored recently. However, only after extensive efficacy and toxicological studies, such strategies might enter clinical trials. Moreover, number of targeted agents was evaluated clinically for chemoprevention of CRC after successful preclinical studies. In this review, we will focus on the targeted drug intervention of selected anti-inflammatory agents, including NSAIDs (aspirin, naproxen, sulindac, celecoxib, and licofelone), and their combinational intervention. Further, we review molecular targets, preclinical trials, efficacies, clinical trials, toxicities and limitations of the aforementioned NSAIDs. Also, we will provide our views and throw light on some future precision medicine approaches for colon cancer chemoprevention.

## 2. Selected Anti-Inflammatory Agents for Colon Cancer Prevention

Of the several drugs tested so far for CRC prevention, substantial evidence for the anti-cancer effects in in vitro, in vivo, epidemiological and clinical studies exist for (1) NSAIDs (aspirin, naproxen, sulindac, celecoxib, licofelone), and (2) Combinational interventions.

### 2.1. NSAIDS for Colon Cancer Prevention

By far, NSAIDs are most widely studied agents for intervention of progression of colon cancer both preclinically and clinically. Aspirin, sulindac, celecoxib and naproxen are extensively studied for chemoprevention of CRC at laboratory, preclinically and clinically. Evidence from epidemiological studies also clearly indicates the chemopreventive effects of NSAIDs against colon cancer. Licofelone is also belongs to novel NSAIDs, which is under various stages of pre-clinical and clinical development as a drug for inflammatory and oncologic diseases.

#### 2.1.1. Aspirin for CRC Prevention

**Figure 1:**
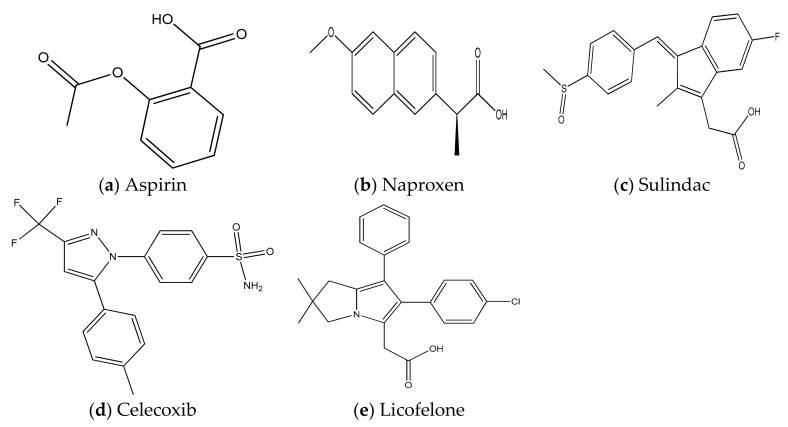
Chemical structures of some of the anti-inflammatory agents with chemopreventive potentials against colorectal cancers. Chemical structures of aspirin (**a**), naproxen (**b**), sulindac (**c**), celecoxib (**d**) and licofelone (**e**).

Aspirin (acetylsalicylic acid), a NSAID, exhibits anti-inflammatory and chemopreventive potentials against several cancers including CRC (Figure 1a) [22]. Large numbers of clinical trials on aspirin for colon cancer are completed and some of them are yet to be completed. Several combinations of aspirin with other chemopreventive agents have been evaluated for prevention of CRC.

##### Molecular Targets

**Figure 2:**
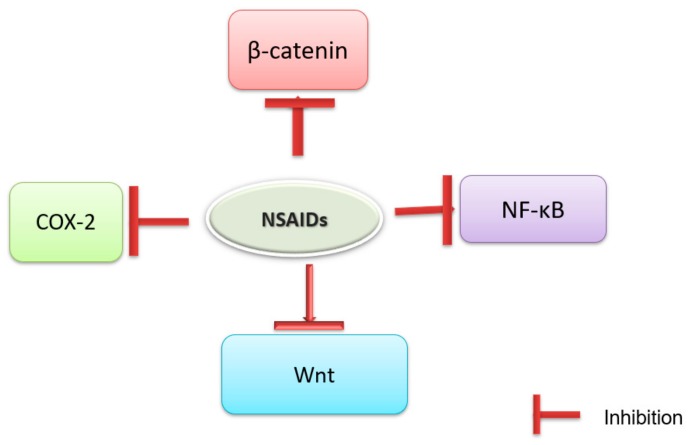
Molecular targets of selected non-steroidal anti-inflammatory drugs (NSAIDs) for colorectal cancer (CRC) prevention.

Mechanistically, aspirin shows its effects by inhibiting the prostaglandin (PG) E2 synthesis by inhibiting cyclooxygenase (COX-1&2) activity elevated in CRC (Figure 2) [23,24,25]. Aspirin inhibits both COX-1 and COX-2 activities [24]. Aspirin exhibits chemopreventive potentials against CRC by modulating wnt/β-catenin signaling pathway (Figure 2) [24]. Regulation of proliferation and induction of apoptosis in colon cancer/normal cells are mediated by through NF-κB, p53 and caspases [26,27,28]. Nitric oxide (NO)-aspirin, a nitric oxide releasing prodrug of aspirin, inhibits the catalytic activity of nitric oxide synthase (NOS)-2, along with two COX-2, as well as down regulated expression of β-catenin and proliferating cell nuclear antigen (PCNA) in colon tumors induced by azoxymethane [29]. Recently, Kagbo-Kue et al. [30] reported that aspirin inhibited the metastasis of colon cancer cells by suppressing the expression of toll-like receptor 4.

##### Pre-clinical Studies

Earlier studies in animal models demonstrated a clear CRC chemoprevention efficacy of aspirin both at initiation and progression stages. The carcinogen (dimethylhydrazine (DMH)/ azoxymethane (AOM))-induced rat colon cancer precursor lesions aberrant crypt foci (ACF) formation and progression were significantly lowered by aspirin [31,32,33,34]. Aspirin was also found to significantly reduce multi-crypt aberrant foci in rats [35]. Pronounced aspirin effects at 60 mg/kg b.w. were seen in the inhibition of distal ACF in DMH induced rat colon carcinogenesis [36]. Dietary daily oral administration of 200 and 400 ppm of aspirin significantly inhibited the incidence and multiplicity of carcinogen induced invasive adenocarcinomas of the colon as well as the size of adenocarcinomas in rats along with reduction of colonic mucosal and tumor PGE_2_ levels. These studies support the notion that ingestion of aspirin may inhibit colon cancer [37]. With regard to colon adenocarcinoma multiplicity, aspirin showed significant inhibitory effect with different treatment regimens with clear dose-response effects [38].

##### Clinical Studies

Several epidemiological studies supported the notion that consumption of aspirin provides protection against many cancers including colorectal cancer [39,40,41]. The results from the Association pour la Prevention par l’Aspirine du Cancer Colorectal (APACC) randomized clinical trial for the prevention of recurrence of colorectal adenoma by aspirin at 160 and 300 mg/day doses showed that daily low-dose aspirin decreased adenoma recurrence significantly at 1 year but not at year 4 [42,43]. Importantly, in a double-blind randomized clinical trial with aspirin at 81 mg and 325 mg per day with 1121 patients, Baron et al. [44] clearly demonstrated that low dose aspirin showed moderate chemopreventive effects against colon tumors. Further, Cole et al. [45] performed a meta-analysis of: Four randomized clinical trials (the Aspirin/Folate Polyp Prevention Study (AFPPS) [44]; the Colorectal Adenoma Prevention Study (Cancer and Leukemia Group B (CALGB) 9270) [46]; the United Kingdom Colorectal Adenoma Prevention (ukCAP) Study [47]; and the Association pour la Prevention par l’Aspirine du Cancer Colorectal (APACC) Study [42]. Cole et al. [45] evaluated aspirin at the doses ranging from 81 mg to 325 mg/day to analyze the adenoma occurrence and advanced lesion occurrence after median follow up of 33 months and observed that aspirin is effective chemopreventive agent in the individuals with these lesions (Table 1). Further, many clinical trials involving aspirin including: Use of alternative dosing strategies are underway to retain its chemoprevention potential while reducing toxicities or side-effects [48]; Aspirin for prevention of postsurgical recurrence and metastasis in asian colorectal cancer patients: a multi-center randomized trial (APREMEC phase III trial) [49]; aspirin in colorectal cancer liver metastases (ASAC) trial to assess the beneficial role of ASA in recurrence of CRC liver metastases and survival [50]; a phase III double-blind placebo-controlled randomized trial of aspirin 80 mg given orally once daily for five years on five year overall survival for stage II and III colon cancer patients and recurrence [51]; finding the best dose of aspirin to prevent Lynch syndrome cancers (CaPP3 Israel) [52]. The final study mentioned determines cancer preventive efficacy of enteric coated aspirin in Lynch syndrome are dose-responsive by comparing overall cumulative incidence rates of the disease after five years in people who took 100 mg, 300 mg, or 600 mg of the same for at least two years. Low-dose of aspirin use improves overall survival of patients with CRC by modulating various biomarkers [53,54,55,56]. But, in a recent study by Gray et al., [57] reported that low-dose aspirin use did not prolong survival rate in patients with CRC. Aspirin at low-dose (80–60 mg/day) reduces the risk of recurrent colorectal adenomas but its effect on advanced adenomas was not clear [58]. A recent study by Wang et al. [10] demonstrated that smoking and body mass index negatively influences of preventive effects of aspirin and other nonsteroidal anti-inflammatory drugs on CRC risk. Finding from the several other ongoing clinical trials including above may provide important data to use aspirin for recurrent and metastatic CRC chemoprevention as well [51,59,60,61].

##### Toxicity and Overcoming Strategies

COX-1 and its products prostaglandins (PGs) involve in maintenance of the integrity of the mucosal epithelium of gastro intestine and their inhibition with the conventional NSAIDs like aspirin causes gastro-duodenal damage, hemorrhage, enteropathy and ulceration as well as cardiovascular side effects [62,63,64].

In this scenario, several research groups have been synthesized NO and/or hydrogen sulfide (H_2_S) releasing aspirin with gastrointestinal (GI) safety, enhanced COX (-1&-2) inhibition as well as better cancer preventive activities than its parental molecule [65]. Previously, we reported that NO-aspirin suppressed both invasive and noninvasive adenocarcinomas of the colon and exhibit GI safety or other any side effects [29].

#### 2.1.2. Naproxen for CRC Prevention

##### Molecular Targets

Naproxen (Figure 1b) shows its effects by inhibiting the PGE_2_ synthesis by inhibiting COX activity in CRC. Historically, naproxen is known to exerts its protective effects as anti-inflammatory agents and lately as anti-cancerous agent due to the similar molecular pathways that exists in inflammatory diseases and cancer. H_2_S-releasing naproxen derivative ATB-346 reduced expression of c-Myc, β-catenin there by exhibits chemopreventive potentials intestinal tumors in *Apc*^Min/+^ mice than naproxen (Figure 2) [66,67].

##### Pre-clinical Studies

A number of studies undertaken over three decades in different animal models strongly establish NSAIDs efficacy in preventing colon cancer, when administered at different stages of disease progression. In a preclinical study naproxen treatment twice-daily for four weeks showed dose-dependent preventive effect on AOM-induced ACF in the colon of mice [67]. Suh et al. [68] showed that chronic dietary administration of 150 ppm of naproxen significantly inhibited AOM-induce colon adenocarcinoma multiplicity in rats. Recently, we have shown that 200 ppm and 400 ppm Naproxen either continuously or one week on/one week off) or 3 weeks on/3 weeks off showed significant inhibitory effects against the AOM-induced colon adenocarcinoma multiplicity in rats [38].

##### Clinical Studies

A phase Ib biomarker clinical trial with naproxen is underway to determine the adverse effects and optimal dose of naproxen in preventing DNA mismatch-repair deficient CRC in patients with Lynch syndrome [69].

##### Toxicity and Overcoming Strategies

Long-term treatment of naproxen causes gastro-duodenal damage, hemorrhage, enteropathy and ulceration (Table 1) [70]. Chattopadhyay et al. [71] synthesized nitric oxide (NO) and/or H_2_S releasing naproxen with GI safety, enhanced COX (-1&-2) inhibition as well as better cancer preventive activities than its parental molecule. H_2_S-releasing naproxen derivative ATB-346 exhibits better intestinal polyp formation as well as GI-sparing effects in *Apc*^Min/+^ mice than naproxen [66,67].

#### 2.1.3. Sulindac for CRC Prevention

Sulindac, a NSAID, is marketed by Merck in the name of Clinoril. Epidemiological and clinical studies demonstrated that sulindac possesses anti-inflammatory and chemopreventive effects against many cancers, including colorectal cancers (Figure 1c and Table 2).

##### Molecular Targets

In the rat colon carcinogenesis, sulindac-induced apoptosis during initiation and promotion stages [78]. Singh et al. [79] studied inhibition of expression of ras-p21 and p53 by sulindac during azoxymethane-induced colon carcinogenesis. Sulindac exhibits anti-inflammatory activity via inhibition of COX-1/COX-2 dependent and -independent effects, modulation of the Wnt, NF-κB and/or STAT3 pathway and activation of adenosine monophosphate-activated protein kinase (AMPK) (Figure 2). Rat colon tumors exposed to sulindac and naproxen individually or in combination with atorvastatin showed significant suppression of PCNA, cyclin D1 and β-catenin and reduced key inflammatory markers, inducible NOS and COX-2, p65, as well as inflammatory cytokines, TNF-α, IL-1β, and IL-4 [68].

##### Pre-clinical Studies

Published studies reported inhibition of both carcinoma induction as well as progression by sulindac in DMH-induced mouse colon carcinogenesis [80,81,82]. However, in the rat, inhibition of AOM-induced adenomas and carcinomas by the agent at 0.04% of diet was accompanied by decreased PGE_2_ synthesis in the colon [82,83]. Sulindac at 10 mg/kg twice daily inhibited the new colon tumor formation and the rate of growth of colon tumors in the rats [81]. Further, we showed that sulindac at 160 and 320 ppm levels when fed during initiation or during promotion/progression stages dose-dependently inhibited the incidence and multiplicity of invasive and noninvasive adenocarcinomas [72]. Also, dietary sulindac (160 and 320 ppm) reduced the colon tumor volume by > 52–62% as compared to the control diet [72]. Further, sulindac at 320 ppm significantly reduced carcinogenesis in the Pirc rat, an APC-driven model of colon carcinogenesis [84].

##### Clinical Studies

Sulindac was studied for its chemopreventive efficacy clinically and was shown to be effective in reducing the number and size of colorectal adenomas in lynch syndrome patients [85]. In another study, Giardiello et al. [86] reported that 43% of the subjects developed adenomas in sulindac treatment groups whereas 55% subjects developed adenomas in placebo treated subjects. Takayama et al. [73] showed that ACF numbers were significantly suppressed by sulindac at 300 mg daily in a randomized trial.

##### Toxicity and Overcoming Strategies

Long-term use of sulindac for FAP is hampered due to its toxic effects. In this scenario, several derivatives of sulindac like nitric oxide (NO) and H_2_S-releasing sulindac, have been synthesized by various research groups and explored for their anti-inflammatory and anticancer activities along with GI safety [87].

#### 2.1.4. Celecoxib for CRC Prevention

Celecoxib (Figure 1d) (Celebrex) is a COX-2 selective NSAID, has been used to treat several inflammation-associated diseases including rheumatoid arthritis, as well as exhibits chemopreventive potentials against CRC (Table 1 and Table 2) [88].

##### Molecular Targets

Celecoxib exhibits chemopreventive potentials against colon cancer by targeting COX-2 [89]. Celecoxib along with imatinib downregulated expressions of COX-2, vascular endothelial growth factor, and NF-κB, as well as increased caspases-3 activity in HT-29 human colorectal cancer cells [90]. Celecoxib inhibits Wnt/β-catenin signaling pathway and its genes products survivin and cyclin D1 there by it shows chemopreventive effects against colon cancer [91,92] (Figure 2).

##### Pre-Clinical Studies

Our laboratory, along with others, has extensively studied the chemopreventive efficacy of celecoxib in pre-clinical animal models of colon carcinogenesis. We first showed that dietary administration of celecoxib at different doses inhibited total ACF induction and crypt multiplicity that strengthened the hypothesis of selective COX-2 inhibition for chemoprevention of colon carcinogenesis [93]. Studies from our laboratory also showed that oral administration of celecoxib inhibited both incidence and multiplicity of colon tumors by about 93% and 97%, respectively [94]. Further, we have shown that administration of celecoxib to the rats during the initiation and post-initiation stages significantly inhibited the incidence as well as the multiplicity of colon adenocarcinomas (*p* < 0.01 to *p* < 0.0001) in a dose-dependent manner and tumor volume (*p* < 0.0002 to *p* < 0.001) [74]. The study results clearly suggested that potential of celecoxib for both primary and secondary chemoprevention. We also showed that celecoxib alone or in combination with other agents reduces both colon and small intestinal tumorigenesis in *Apc*^Min/+^ mouse model [95]. Jacoby et al. demonstrated that celecoxib is effective for the prevention of intestinal adenomas in the mouse model of adenomatous polyposis [96].

##### Clinical Studies

Celecoxib, a select COX-2 inhibitor, at 200 mg or 400 mg twice daily was found to be very effective for the prevention of colon adenomas in the randomized clinical trials but caused potential cardiovascular events particularly for patients with preexisting atherosclerotic heart disease thus limiting its advancement [76,97,98]. Bertagnolli et al. [97] reported that patients receiving celecoxib exhibited chemopreventive effects by decreasing the cumulative incidence of advanced adenomas over 5 years. In the PreSAP Trial Investigators, Arber et al. [99] showed that use of 400 mg of celecoxib once daily significantly reduced the occurrence of colorectal adenomas within three years after polypectomy. Although very effective, select COX-2 inhibitors usage is hindered due to their potential side-effects. In a clinical trial, Wang et al. [75] examined adenomas using immunohistochemistry to assess expression of COX-2 before treatment. Celecoxib-treatment caused substantial reduction in adenomas in which COX-2 expression is more.

##### Toxicity and Overcoming Strategies

Celecoxib exhibits cardiovascular side effects [100]. It may not be used in Individuals at high risk for cardiovascular events. Celecoxib can be studied by intermittent dosing approaches to test if efficacy is maintained while cardio-toxicity is minimized. New strategies of chemoprevention are urgently needed in order to effectively utilize NSAIDs by overcoming their unwanted side-effects.

#### 2.1.5. Licofelone for CRC Prevention

Licofelone (Figure 1e), a dual COX and 5-LOX dual inhibitor, has been clinically evaluated for osteoarthiritis [101]. Accumulating evidences suggested that licofelone exhibited chemopreventive potentials against several cancers, including colorectal cancers.

##### Molecular Targets

Licofelone significantly decreased COX and 5-LOX activities, reduced proliferating cell nuclear antigen expression (70%, *p* < 0.0001), suppressed of serum triglycerides (71–83%, *p* < 0.0001), and decreased inflammatory cytokines in intestinal tumors of *Apc*^Min/+^ mice [77]. Licofelone-induced apoptosis in a dose- and time-dependent manner in HCA-7 colon cancer cells via intrinsic pathway [102]. Licofelone caused loss in mitochondrial membrane potential, cytochrome c release, activation of caspase-9, and 3 and poly-(ADP-ribose) polymerase-1 cleavage. In addition, licofelone-induced the cleavage of p21Bax into p18Bax [102].

##### Preclinical Trials

Licofelone significantly suppressed total intestinal tumor multiplicity and size in both male and female *Apc*^Min/+^ mice [77] (Table 2). Dietary licofelone at 150 or 300 ppm for 14 weeks showed substantial inhibition of intestinal tumors 72% and 100% inhibition of colonic tumors, respectively [77]. Overall, results of these preclinical animal studies on licofelone are encouraging and warranted its clinical trials for CRC prevention. Licofelone has been tested in clinical trial for GI ulceration however it is yet to be evaluated for chemopreventive efficacy against CRC.

##### Toxicities and Limitations

Licofelone shows GI safety profile compared to other NSAIDs and also suitable for the long-term treatment of inflammatory and oncologic diseases (Table 1) [90].

### 2.2. Combinational Intervention

Combination intervention has been in use in order to achieve higher efficacies and also to reduce the doses of individual drugs to overcome drug toxicities and drug resistance. Several preclinical studies till date are reported on many combination agents for colon cancer prevention using different mice and rat models. Here, we discuss only those combinations which moved into clinical trials. Since particular combination interventions are highlighted, respective molecular targets have already been referred in earlier sections.

Recent studies have shown that both aspirin and metformin are inhibitors of mTOR/S6K1 and activates AMPK. Petrera et al. [103] are conducting ASAMET: Randomized, placebo-controlled, double-blind, 2 × 2 biomarker trial of aspirin and metformin to evaluate the efficacy of these agents alone and in combinational effect on surrogate biomarkers of colorectal carcinogenesis. The primary endpoint is the change, means the difference between pre- as well as post-treatment expression of NF-κB in the unaffected mucosa of proximal and distal colon obtained by multiple biopsies in paired colonoscopies one year apart and with additional biomarkers analyses (pS6K, p53, β-catenin, PI3K) [103]. A population-based study by De Monte et al. [104] showed improved survival for type II diabetes patients with stage II and III CRC with aspirin and metformin. Several researchers have been explored combination of aspirin and calcium as one of the cost effective and low-risk strategies with colonoscopy for primary prevention of CRC [105].

In a double-blind, placebo-controlled, randomized trial (phase II) by Samadder et al. [106] reported that combination treatment with sulindac and erlotinib significantly reduced colorectal polyp burden after 6 months of treatment as compared with placebo. In a preclinical study by Chang et al. [107] demonstrated that treatment with combination of sulindac and atorvastatin decreased the multiplicity of colon adenomas in *Apc*^Min/+^-FCCC mice already with adenomas.

Sulindac along with difluoromethylornithine (DFMO) showed significant colon cancer prevention activity [108]. A successful combination chemoprevention drugs DFMO at the dose of 500 mg and sulindac at the dose of 150 mg once daily was significantly effective for prevention of adenomas in 3 years randomized placebo controlled clinical trial [109]. The results obtained in the Meyskens et al. [109] study involving 375 patients, the DFMO plus sulindac drug combination reduced the overall incidence of adenoma recurrence by 70%, from 41% in the placebo group to 12% with the combination treatment [109,110]. However, ≥ grade 3 adverse events were seen in 11% patients in the combination treatment group compared to 8.2% patients in placebo group (*p* = 0.35) [109]. This is a major success in utilizing lower doses of DFMO and sulindac for chemoprevention of colon cancer by combination intervention [110]. In line with these experiments, currently a new clinical trial is underway in the FAP patients with 750 mg DFMO and 150 mg sulindac individually and in combination in a randomized double-blind phase III trial [111,112]. The rationale for these studies stems from the fact that polyamines are greatly inhibited by DFMO in the colonic mucosa and that sulindac exerts its effects by modulation of wnt pathway and by enhancing the polyamine catabolism by upregulating transport genes *PPARγ* and *SAT* that the combination might be more effective [111].

A recent international randomized trial with 112 FAP patients with celecoxib (400 mg twice a day) alone or in combination with DFMO (0.5 g/m^2^/day rounded down to the nearest 250 mg dose) did not show a significant difference in adenoma count [113]. However, there was no evidence of DFMO associated ototoxicity or celecoxib associated cardiovascular toxicity [113].

## 3. Conclusions and Perspectives

Unresolved questions with regard to efficacy, safety, optimal treatment regimen, and mechanism of action limit the clinical utility of NSAIDs for the prevention of CRC in patients. A number of drugs already exists that have been widely studied across different diseases that target several similar mechanistic targets. Most targeted therapeutic drugs also exist with enormous preclinical and clinical data. Above mentioned agents and their combinations possess activities against different diseases. Major hurdles with these drugs to move forward to clinic are their lack of significant safety where the bar is quite high for chemoprevention compared to chemotherapy. Currently, the focus is changing on the way cancer treatment towards different precision medicine strategies. Drugs that are developed for specific targets or mutations are now being explored across organ site cancers that have specificity for the targets. For example, all those cancers having specific mutations can be targeted using a drug specifically designed for that mutation. In a similar way, various drugs may be repurposed for cancer chemoprevention from existing drugs studied or approved for different diseases/cancers for therapy by minimizing toxicity while maintain efficacy.

Certain drugs, although very effective, might show other unwanted side effects. COX-2 inhibitors are good examples that showed great effects against colon cancer at preclinical and clinical trials. However, they pose a risk for cardiovascular events. In this case, celecoxib can be studied by intermittent dosing approaches to test if efficacy is maintained while cardiotoxicity is minimized. These drugs may not be used in Individuals at high risk for cardiovascular events. Further, mechanistically, developing agents that target downstream enzymes that specifically inhibit microsomal prostaglandin E synthase (mPGES)-1 thereby PGE_2_ production overcoming cardio-toxicity by protecting PGI_2_ is an alternative approach. In this direction, we have developed LFA9, with dual 5-LOX and mPGES-1 inhibitor as a safer colon cancer chemopreventive that was observed to be safe and highly efficacious in preclinical animal models [114,115]. Measures to overcome toxicities of known drugs are very important. For example, NSAIDs caused GI toxicity can be overcome by following alternative strategies like intermittent dosing of the agents to alleviate the toxic effects or by using very low doses in combination with other drugs might be considered. We tested known NSAIDs naproxen and aspirin to evaluate their chemopreventive efficacy by short-term frequent intermittent dosing approaches using preclinical animal models and observed that intermittent dosing approach provided efficacy similar to continuous dosing approach [38]. Importantly, NSAIDs must be used in individuals at younger age who tend show lower GI toxicity compared to very old patients. For agents like DFMO, strategies must be designed by using lower doses and combining with other agents to limit oto-toxicity. Several preclinical studies are already existing to support the efficacy of DFMO with other agent combinations; however, oto-toxicity needs to be evaluated before moving towards clinical trials.

Other approaches like use of in-silico technologies, drug alternatives must be designed improve specificity towards the targets leads to overcome side-effects. Also, a search into the already existing natural or synthetic agents that are safe for human consumption must be undertaken through in-silico modeling to identify novel and safer colon cancer chemopreventive agents for specific targets (drug repurposing). If mutation-targeted agents are being developed, they must be first evaluated in appropriate animal models with those mutations and later clinical trials with patients showing those mutations must be done. Drug development against *KRAS*, *BRAF* or *PIK3CA* mutations and microsatellite instability (MSI) may be steps towards developing precision medicine strategies for patients with these mutations. However, an important consideration towards the mutational status at the early stage of cancer development is needed to initiate respective intervention and followed up by additional screenings at later stages if drug resistance emerges due to new mutations that develop which calls for additional drug interventions.

FAP-associated desmoid tumors are sometimes life threatening and difficult to treat [116,117]. Due to limitations to surgery for FAP-related desmoid tumors, the use of NSAIDs can be an effective preventive and therapeutic strategy [116,117]. In addition, combination of NSAIDs with estrogen receptor modulators can be an effective strategy for prevention and/or treatment of patients with FAP-related desmoid tumors [118].

Of late, immunoprevention is gaining significant importance in lieu of the success with HPV vaccines against cervical cancer. In this context, effective agents against CRC, aspirin and other NSAIDs, metformin and DFMO are being explored for their immunomodulatory effects. Also, efforts are underway for the development of vaccines against CRC especially for those related to MSI. Improved understanding of immune aspects at early stages of premalignant and primary tumor microenvironment and agent’s immunomodulatory effects alone or in combination with vaccines might be a potential future for effective CRC chemoprevention.

Appropriate dietary regiments, nutritional supplements, and exercise may be used in conjunction with chemopreventive agents to personalize considering each individual needs to attain maximum benefit with minimum risks and side-effects. Chemoprevention of colon cancer towards precision medicine approach is long-way to go?however, these strategies might be initially tested using appropriate animal models. First and foremost, a detailed pre-cancer and cancer atlas in animal models that can be compared to humans at different stages of colon cancer development must be developed before undertaking any such precision medicine studies in preclinical animal models which will pave the way towards clinical trials.

## Figures and Tables

**Table 1:** Comparison and similarities among selected NSAIDs in drug development for CRC.

Characteristic	Aspirin	Naproxen	Sulindac	Celecoxib
Current stage of drug development for CRC	Pre-clinical and clinical	Pre-clinical and clinical	Pre-clinical and clinical	Pre-clinical and clinical
Clinically relevant and effective doses	81 mg to 325 mg/day	220 to 440 mg/day	300 mg/day	<800 mg/day
Molecular Targets	COX-1&2, wnt, β-catenin, NF-κB, p53, toll-like receptor 4, NOS-2 and caspases	COX, c-Myc, NF-κB and β-catenin	p21, p53, Wnt, NF-κB, STAT3, TNF-α, IL-1β, IL-4, AMPK, PCNA, cyclin D1, β-catenin, inducible NOS, COX-2 and p65	COX-2, VEGF, NF-κB, caspases-3, Wnt, β-catenin, survivin and cyclin D1
Toxicity	Gastro-duodenal damage, hemorrhage, enteropathy and ulceration as well as cardiovascular side effects	Gastro-duodenal damage, hemorrhage, enteropathy and ulceration.	Gastro-duodenal damage, hemorrhage, enteropathy and ulceration	Cardiovascular side effects

**Table 2:** Chemopreventive potentials of selected anti-inflammatory agents against colon cancer.

Type of Agent	Experimental Type	Observation	References
Aspirin	Pre-clinical	Colon cancer precursor lesions aberrant crypt foci (ACF) formation and progression were significantly lowered in chemical carcinogens (DMH/AOM)-induced CRC in rats.	[31,32,33,34]
	Clinical trail	Aspirin at 160 and 300 mg/day doses showed that daily low-dose aspirin decreased adenoma recurrence significantly at 1 year but not at year 4.	[42,43]
	Clinical data analysis	Aspirin at the doses ranging from 81 mg to 325 mg/day to analyze the adenoma occurrence and advanced lesion occurrence after median follow up of 33 months and observed that aspirin is effective chemopreventive agent in the individuals with these lesions.	[54]
	Clinical trials	Low-dose of aspirin use improves overall survival of patients with CRC by modulating various biomarkers.	[53,54,55,56]
Naproxen	Pre-clinical	Administration of 200 and 400 ppm of Naproxen inhibited colon adenocarcinoma multiplicity	[38]
	Pre-clinical	Dose-dependent prevention of AOM-induced ACF in the colon of mice by naproxen treated twice-daily for 4 weeks.	[67]
	Pre-clinical	H_2_S-releasing naproxen derivative ATB-346 exhibits better intestinal polyp formation as well as GI-sparing effects in *Apc*^Min/+^ mice than naproxen.	[66]
	A phase Ib clinical trial	In progress to determine the adverse effects and optimal dose of naproxen in preventing DNA mismatch-repair deficient colorectal cancer in patients with Lynch syndrome.	[69]
Sulindac	Pre-clinical	Sulindac (160 and 320 ppm) reduced tumor volume of the colon by > 52–62% as compared to the control.	[72]
	Clinical trail	ACF numbers were significantly suppressed by sulindac at 300 mg daily in a randomized trial.	[73]
Celecoxib	Pre-clinical	Initiation and post-initiation stages significantly inhibited the incidence as well as the multiplicity of colon adenocarcinomas in rats.	[74]
	Clinical trail	Celecoxib-treatment caused substantial reduction in adenomas in which COX-2 expression is more.	[75]
	Clinical study	800 mg daily dose showed substantial adenoma prevention, but this dose caused cardiovascular toxicity.	[76]
Licofelone	Pre-clinical	Licofelone significantly dose-dependently inhibited size as wells as multiplicity of the intestinal tumors in both male and female *Apc*^Min/+^ mice.	[77]

## Abbreviations

ACF	Aberrant Crypt Foci
APC	Adenomatous Polyposis Coli
AMPK	Adenosine Monophosphate-activated Protein Kinase
AOM	Azoxymethane
CRC	Colorectal Cancers
COX	Cyclooxygenases
DFMO	Difluoromethylornithine
DMH	Dimethylhydrazine
FAP	Familial Adenomatous Polyposis
GI	Gastrointestinal
H_2_S	Hydrogen Sulfide
NOS	Nitric Oxide Synthase
NSAIDs	Non-Steroidal Anti-inflammatory Drugs
MPGES	Microsomal Prostaglandin E Synthase
PCNA	Proliferating Cell Nuclear Antigen
PGs	Prostaglandins
